# Association between waist-to-height ratio and cognitive impairment among community-dwelling elderly adults in Laoshan, Qingdao: a cross-sectional study

**DOI:** 10.3389/fnut.2026.1848267

**Published:** 2026-07-13

**Authors:** Fang Yuan, Yi Zhou, Xuelian Yl, Yi Zhang, Bing Liang

**Affiliations:** 1 Department of Geriatrics, Qilu Hospital of Shandong University, Qingdao, China; 2 Department of Ultrasound, Rizhao Traditional Chinese Medicine Hospital, Rizhao, China; 3 Community Health Service Center of Laoshan District, Qingdao, China; 4 Department of Neurology, Rizhao Central Hospital, Rizhao, China; 5 Department of Neurology, Women and Children's Hospital, Qingdao University, Qingdao, China

**Keywords:** cognitive impairment, community-based cohort, elderly population, non-linear association, northern coastal China, waist-to-height ratio

## Abstract

**Objective:**

This study investigated the association between waist-to-height ratio (WHtR) and cognitive impairment in community-dwelling elderly individuals in Laoshan district, Qingdao, China, focusing on the dose-response relationship.

**Methods:**

We conducted a community-based cross-sectional study, enrolling 3,093 elderly residents aged 60–80 years from urban and rural areas in Laoshan District. Data on demographic characteristics, lifestyle factors, medical comorbidities, and standardized anthropometric measurements (height, weight, waist circumference) were collected. WHtR was calculated as waist circumference (cm) divided by height (cm) and categorized into quartiles. Cognitive function was assessed using the Chinese version of the montreal cognitive assessment basic (MoCA-B). Univariate and multivariate logistic regression analyses were performed to examine the independent association between WHtR and cognitive impairment risk. Restricted cubic spline (RCS) regression and breakpoint analysis assessed the non-linear relationship.

**Results:**

The association was evaluated using the third WHtR quartile (Q3: 0.5823 ≤ WHtR < 0.6049) as the reference. Multivariate logistic regression revealed that, after adjusting for major comorbidities, the Q1 group (WHtR < 0.5556), and Q4 group (WHtR ≥ 0.6049) showed significantly higher risks of cognitive impairment (odds ratios [OR] = 1.93 and 2.79, respectively; both *P* < 0.001). The Q2 group (0.5556 ≤ WHtR < 0.5823) also showed an increased risk (OR = 1.27, *P* = 0.045). RCS analysis confirmed a significant non-linear association (*P* for non-linearity < 0.001), with a critical breakpoint at WHtR × 100 = 59.394 (95% CI:59.244 to 59.544); values above this threshold were linked to a significantly increased risk of cognitive impairment (OR = 1.208, 95% CI: 1.090–1.339, *P* = 0.0003).

**Conclusion:**

This study demonstrates a significant non-linear dose-response relationship between WHtR and cognitive function in older adults. Excessive abdominal adiposity beyond a critical threshold negatively impacts cognitive function. Weight management programs for older adults should emphasize maintaining optimal body weight to support cognitive health.

## 1 Introduction

Cognitive impairment, a progressive age-related neurocognitive impairment, is characterized by deficits in memory, executive function, and language abilities. This condition significantly diminishes quality of life and increases the risks of disability and mortality among the elderly (1). The accelerated global aging population has led to a rising prevalence of cognitive impairment, imposing substantial socioeconomic and public health burdens worldwide (2). Therefore, identifying modifiable risk factors and effective early screening indicators is critical for primary prevention and targeted intervention against age-related cognitive impairment in this population (3, 4). Central obesity is widely recognized as a significant modifiable determinant of cognitive decline. It triggers chronic low-grade inflammation, insulin resistance, and damage to cerebral microvascular endothelium, disrupting synaptic plasticity, accelerating neuronal apoptosis, and ultimately contributing to cognitive deterioration (5–7). The waist-to-height ratio (WHtR) is considered a more reliable indicator of central obesity compared to body mass index (BMI) and waist circumference (WC) because it accounts for height differences and demonstrates superior efficacy in assessing abdominal adiposity across diverse ethnic and age groups (8).

Current epidemiological evidence regarding the association between WHtR and cognitive performance is inconsistent. An analysis of the U.S. National Health and Nutrition Examination Survey (NHANES) identified a significant inverse linear relationship between WHtR and cognitive function in older adults, with an inverted non-linear association observed exclusively among female participants (9). In contrast, a national longitudinal study in China found that both low and high WHtR levels were associated with an increased risk of cognitive impairment in elderly individuals (10).

In light of these findings, this community-based cross-sectional study enrolled 3,093 elderly residents from urban and rural communities in Laoshan district to explore the association between WHtR and cognitive impairment. The study aims to verify the dose-response relationship and identify the critical clinical cutoff for WHtR associated with cognitive impairment risk. Ultimately, this research seeks to provide evidence-based anthropometric indicators for early screening and targeted prevention of cognitive impairment among elderly populations in northern coastal China.

## 2 Methods

### 2.1 Study participants

This cross-sectional study was approved by the Ethics Committee of the Qilu Hospital of Shandong University (Qingdao) [No. 2023056]. All participants provided written informed consent prior to enrollment, in accordance with the ethical principles outlined in the Declaration of Helsinki. Participants were excluded based on the following criteria: individuals aged 60–80 years with incomplete or extreme anthropometric measurements (BMI, WC, WHtR), missing cognitive assessments, or incomplete covariate records. After applying these criteria, the final analytical sample comprised 3,093 eligible participants.

**Figure 1:**
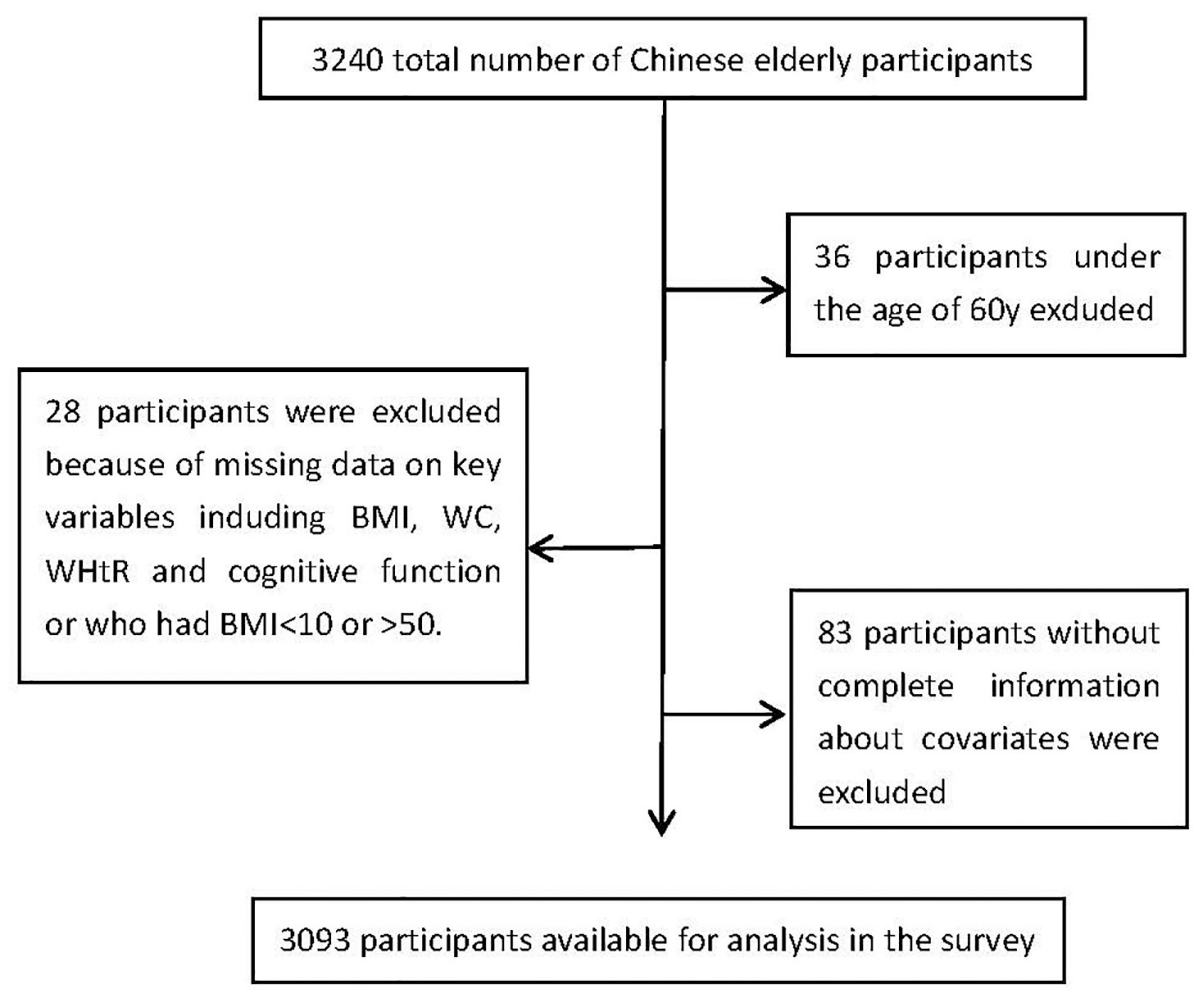
Participant selection proceness flowchart.

This study recruited 3,240 elderly residents aged 60–80 years from 12 urban and eight rural communities in the Laoshan district of Qingdao, northern China, between October 2022 and December 2023. Participants were selected from 42 randomly chosen communities out of a total of 165 in the district. All older adults residing in these selected communities visited their respective community health service centers to complete the survey. For this cross-sectional analysis, baseline data from the Laoshan community-dwelling cohort were examined. Participants meeting any of the following exclusion criteria were excluded from the analysis: age below 60 years, incomplete or extreme anthropometric measurements (BMI, WC, or WHtR), missing cognitive evaluations, or incomplete demographic and health records. After applying these criteria, the final analytical sample included 3,093 eligible participants (Figure 1).

Inclusion Criteria: (1) Age between 60 and 80 years. (2) Long-term residency in Laoshan district for at least five consecutive years. (3) Ability to cooperate with standardized anthropometric and cognitive function assessments. (4) Complete clinical and demographic data without significant missing values. Exclusion Criteria: (1) Severe psychiatric disorders or communication deficits hindering assessment. (2) History of acute cerebrovascular events within the past 3 months. (3) Diagnosis of malignant tumors, end-stage organ failure, or other severe systemic comorbidities. (4) Missing core data related to WHtR or cognitive function scores.

### 2.2 Data collection and standardized assessments

#### 2.2.1 BMI, WC, and WHtR

During the investigation, trained interviewers measured participants' body weight (in kilograms) and height (in centimeters) following established protocols. Body mass index (BMI) was calculated by dividing body weight by the square of height (kg/m^2^). Participants were instructed to stand upright, relax their abdomen, and breathe calmly during measurements. Waist circumference (WC) was measured using a flexible tape measure at the midpoint between the lower rib margin and the iliac crest. Each measurement was taken twice, and the average value was used for analysis. The waist-to-height ratio (WHtR) was computed as waist circumference (cm) divided by height (cm). For subgroup analysis, WHtR was stratified into four quartiles: Q1 (0.43 ≤ WHtR < 0.5556), Q2 (0.5556 ≤ WHtR < 0.5823), Q3 (0.5823 ≤ WHtR < 0.6049), and Q4 (0.6049 ≤ WHtR ≤ 0.74)(11).

#### 2.2.2 Cognitive function

Cognitive function was assessed using the Chinese version of the montreal cognitive assessment basic (MoCA-B), a validated 30-point screening tool designed for elderly Chinese populations (12). The optimal cutoff scores for screening cognitive impairment were established at 19 for individuals with no more than 6 years of education, 22 for those with 7–12 years, and 24 for individuals with more than 12 years of education. The MoCA-B evaluates several cognitive domains, including orientation, memory, attention, calculation, language, and construction ability. It has been shown to effectively identify mild cognitive impairment (MCI) in Chinese elderly adults. The assessment was administered by trained evaluators following standardized protocols.

#### 2.2.3 Assessment of covariates

Covariates included gender, age (years), marital status, educational level, place of residence, smoking status, drinking status, hypertension, diabetes, osteoporosis, and history of cerebrovascular or cardiovascular diseases. Educational level was measured as a continuous variable representing years of schooling. Participants were classified as smokers or drinkers if they reported current smoking or drinking, regardless of frequency or quantity. Medical history was assessed through self-reporting, asking participants if they had been diagnosed with hypertension, diabetes, or cerebrovascular diseases; those who responded “yes” were considered to have these conditions.

A standardized structured questionnaire was administered by trained medical personnel to collect comprehensive data, including demographic characteristics, lifestyle factors, and medical histories of chronic comorbidities. Educational attainment was classified into five categories: illiteracy (0 years of schooling), primary education (1–6 years), junior high school (7–9 years), senior high school (10–12 years), and college or higher education (>12 years).

### 2.3 Statistical analysis

Data management and statistical analyses were conducted using R software (version 4.2.1). Normally distributed continuous variables are presented as means ± standard deviations. Intergroup differences were assessed using independent samples *t*-tests. Categorical variables are expressed as frequencies and percentages, with intergroup comparisons performed using the X^2^ test. Univariate logistic regression analysis was conducted to identify potential risk factors associated with cognitive impairment.

Multivariate logistic regression models evaluated the independent association between WHtR quartiles and the risk of cognitive impairment, employing three sequential adjustment models: Model 1 (crude model, unadjusted); Model 2 (adjusted for gender, age, educational level, BMI, smoking status, and alcohol consumption); and Model 3 (further adjusted for hypertension, stroke/transient ischemic attack, coronary heart disease, osteoporosis, hyperlipidemia, and type 2 diabetes mellitus).

To characterize the non-linear relationship between continuous WHtR and cognitive impairment risk, restricted cubic spline regression was employed, with knot positions predefined at the 5th, 25th, 50th, 75th, and 95th percentiles of the WHtR distribution. Additionally, segmented breakpoint analysis was conducted using the segmented package in R software to identify abrupt changes in the exposure-risk relationship. The optimal breakpoint was determined by minimizing the residual sum of squares, thereby identifying the clinically relevant WHtR threshold at which its correlation with cognitive impairment risk varied significantly. A two-tailed *P* value < 0.05 was considered statistically significant for all analyses.

## 3 Results

### 3.1 Baseline characteristics of study participants

A total of 3,093 participants, with a mean age of 68.6 ± 5.7 years, were enrolled in this study; 1,724 (55.7%) were female. The mean BMI of the participants was 25.2 ± 2.9 kg/m^2^. Cognitive impairment was determined based on MoCA-B scores, indicating that 1,700 (54.96%) participants exhibited signs of cognitive impairment (Table 1).

**Table 1:** The characteristics of participants according to quartiles of WHtR.

Variables	Total (*n* = 3093)	*Q*1 (*n* = 772)	*Q*2 (*n* = 763)	*Q*3 (*n* = 782)	*Q*4 (*n* = 776)	*p*
Gender, *n* (%)						<0.001
Female	1724 (55.7)	351 (45.5)	371 (48.6)	517 (66.1)	485 (62.5)	
Male	1369 (44.3)	421 (54.5)	392 (51.4)	265 (33.9)	291 (37.5)	
Age, Mean ± SD	68.6 ± 5.7	69.8 ± 6.3	68.4 ± 5.5	67.5 ± 5.4	69.0 ± 5.2	<0.001
Edu, *n* (%)						<0.001
No former education	281 (9.1)	85 (11)	54 (7.1)	38 (4.9)	104 (13.4)	
Primary	1251 (40.4)	307 (39.8)	281 (36.8)	320 (40.9)	343 (44.2)	
Junior high school	1561 (50.5)	380 (49.2)	428 (56.1)	424 (54.2)	329 (42.4)	
Body mass index, Mean ± SD	25.2 ± 2.9	22.0 ± 1.5	24.2 ± 1.4	25.8 ± 1.4	28.8 ± 1.7	<0.001
Smoking, *n* (%)						<0.001
Smoking	387 (12.5)	152 (19.7)	99 (13)	72 (9.2)	64 (8.2)	
Former	113 (3.7)	38 (4.9)	28 (3.7)	17 (2.2)	30 (3.9)	
Never	2593 (83.8)	582 (75.4)	636 (83.4)	693 (88.6)	682 (87.9)	
Drinking, *n* (%)						<0.001
Drinking	470 (15.2)	140 (18.1)	136 (17.8)	88 (11.3)	106 (13.7)	
Former	69 (2.2)	28 (3.6)	14 (1.8)	10 (1.3)	17 (2.2)	
Never	2554 (82.6)	604 (78.2)	613 (80.3)	684 (87.5)	653 (84.1)	
Hypertension, n (%)						<0.001
No	1280 (41.4)	380 (49.2)	353 (46.3)	309 (39.5)	238 (30.7)	
Yes	1813 (58.6)	392 (50.8)	410 (53.7)	473 (60.5)	538 (69.3)	
Stroke TIA, *n* (%)						0.76
No	3047 (98.5)	760 (98.4)	752 (98.6)	773 (98.8)	762 (98.2)	
Yes	46 (1.5)	12 (1.6)	11 (1.4)	9 (1.2)	14 (1.8)	
Coronary heart disease, *n* (%)						<0.001
No	2693 (87.1)	649 (84.1)	688 (90.2)	703 (89.9)	653 (84.1)	
Yes	400 (12.9)	123 (15.9)	75 (9.8)	79 (10.1)	123 (15.9)	
Osteoporosis, *n* (%)						<0.001
No	2723 (88.0)	654 (84.7)	690 (90.4)	721 (92.2)	658 (84.8)	
Yes	370 (12.0)	118 (15.3)	73 (9.6)	61 (7.8)	118 (15.2)	
Hyperlipidemia, *n* (%)						<0.001
No	2600 (84.1)	681 (88.2)	673 (88.2)	658 (84.1)	588 (75.8)	
Yes	493 (15.9)	91 (11.8)	90 (11.8)	124 (15.9)	188 (24.2)	
Diabetes, *n* (%)						0.002
No	2311 (74.7)	600 (77.7)	593 (77.7)	568 (72.6)	550 (70.9)	
Yes	782 (25.3)	172 (22.3)	170 (22.3)	214 (27.4)	226 (29.1)	
MOCA-B, *n* (%)						<0.001
	1393 (45.0)	310 (40.2)	404 (52.9)	456 (58.3)	223 (28.7)	

WHtR, waist height to ratio. WHtR was classified as Q1 (WHtR < 0.5556), Q2 (0.5556 ≤ WHtR < 0.5823), Q3 (0.5823 ≤ WHtR < 0.6049), and Q4 (WHtR ≥ 0.6049).

Compared to participants with normal cognitive function, those in the cognitive impairment group were older, more likely to be female, and had lower educational attainment. Additionally, this group demonstrated a higher prevalence of comorbid conditions, including hypertension, diabetes, stroke, coronary heart disease, osteoporosis, and hyperlipidemia. No significant difference in BMI was observed between the two groups (*P* = 0.052).

### 3.2 Influencing factors of cognitive impairment

Participants were categorized into four groups based on WHtR quartiles (Table 2), systematically investigating the association between WHtR and cognitive impairment. Key findings include:

**Demographic factors**: Being male (OR = 0.70, 95% CI: 0.61–0.81) and higher educational attainment (Edu1: OR = 0.16; Edu2: OR = 0.07) were protective factors against cognitive impairment. In contrast, advancing age emerged as a significant risk factor (OR = 1.11, 95% CI: 1.09–1.12).
**Lifestyle factors**: Current drinking status (Drinking_Stat3, OR = 1.22, 95% CI: 1.00–1.48) was significantly associated with an increased risk of cognitive impairment, whereas smoking status did not show a significant correlation (*P* > 0.05).
**Comorbid Conditions**: Presence of comorbidities was strongly linked to cognitive impairment. Notable associations included hypertension (OR = 1.36), stroke/transient ischemic attack (TIA; OR = 2.35), coronary heart disease (OR = 1.60), osteoporosis (OR = 2.76), hyperlipidemia (OR = 1.43), and type 2 diabetes mellitus (OR = 1.30). Among these, osteoporosis demonstrated the strongest association. Among these, osteoporosis demonstrated the strongest association with cognitive impairment.
**Core indicators**: Each unit increase in WHtR (multiplied by 100) was associated with significantly elevated risk of cognitive impairment (OR = 1.06, 95% CI: 1.04–1.08, *P* < 0.001). The association between BMI and cognitive impairment approached significance (OR = 1.02, 95% CI: 1.00–1.05, *P* = 0.052).

**Table 2:** The correlation between clinical characteristics and cognitive impairment.

Characteristics	OR 95%CI	*P* value
Gender		
Female	Reference	
Male	0.70 (0.61–0.81)	<0.001
Age	1.11 (1.09–1.12)	<0.001
Education		
No former education	Reference	
Primary	0.16 (0.11–0.25)	<0.001
Junior high school	0.07 (0.05–0.11)	<0.001
Body mass index	1.02 (1.00–1.05)	0.052
Smoking	Reference	
Former	1.31 (0.86–2.01)	0.209
Never	1.06 (0.86–1.31)	0.589
Drinking	Reference	
Former	1.61 (0.96–2.71)	0.071
Never	1.22 (1.00–1.48)	0.049
Hypertension		
No	Reference	
Yes	1.36 (1.17–1.57)	<0.001
Stroke		
No	Reference	
Yes	2.35 (1.21–4.55)	0.011
Coronary heart disease		
No	Reference	
Yes	1.60 (1.28–1.99)	<0.001
Osteoporosis		
No	Reference	
Yes	2.76 (2.16–3.54)	<0.001
Hyperlipidemia1		
No	Reference	
Yes	1.43 (1.17–1.74)	<0.001
Diabetes		
No	Reference	
Yes	1.30 (1.10–1.53)	0.002
WHtR100	1.06 (1.04–1.08)	<0.001

WHtR, waist height to ratio; CI, confidence interval; OR, odds ratio.

### 3.3 Associations of WHtR with cognitive impairment

The analysis evaluated WHtR as a continuous variable, using the third WHtR quartile (Q3) as the reference group across three progressively adjusted regression models (Table 3).

**Crude model (model 1)**: Compared with the reference group, the risk of cognitive impairment was significantly elevated in the first quartile (Q1; OR = 2.08, 95% CI: 1.70–2.55) and the fourth quartile (Q4; OR = 3.47, 95% CI: 2.81–4.28) (both *P* < 0.001). The second quartile (Q2) also indicated a moderate increase in risk (OR = 1.24, *P* = 0.034).
**Partially adjusted model (model 2)**: After adjusting for age, sex, educational attainment, BMI, smoking status, and alcohol consumption, the risk of cognitive impairment remained significantly elevated in Q1 (OR = 1.96) and Q4 (OR = 2.86) (both *P* < 0.001). The association in the Q2 group also remained statistically significant (OR = 1.27, *P* = 0.044).
**Fully adjusted model (model 3)**: Following further adjustment for major comorbidities, including hypertension, diabetes mellitus, stroke, and coronary heart disease, the associations slightly weakened; however, the Q1 and Q4 groups continued to demonstrate significantly higher risks of cognitive impairment (OR = 1.93 and 2.79, respectively; both *P* < 0.001). The association in the Q2 group remained stable (OR = 1.27, *P* = 0.045).

**Table 3:** Association between WHtR cognitive impairment.

	OR(95%CI),p						
Variable	*n*, total	Model1		Model2		Model3	
WHtR100	3093	1.06 (1.04–1.08)	<0.001	1.14 (1.08–1.19)	<0.001	1.14 (1.08–1.2)	<0.001
Quartile							
Q1 (WHtR < 0.5556)	772.0	2.08 (1.7–2.55)	<0.001	1.96 (1.45–2.66)	<0.001	1.93 (1.42–2.62)	<0.001
Q2 (0.5556 ≤ WHtR < 0.5823)	763.0	1.24 (1.02–1.52)	0.034	1.27 (1.01–1.61)	0.044	1.27 (1.01–1.61)	0.045
Q3 (0.5823 ≤ WHtR < 0.6049)	782.0	1(Ref)		1(Ref)		1(Ref)	
Q4 (WHtR ≥ 0.6049)	776.0	3.47 (2.81–4.28)	<0.001	2.86 (2.17–3.78)	<0.001	2.79 (2.11–3.69)	<0.001

WHtR, Waist height to ratio; CI, confidence interval; OR, Odds ratio.

Model 1 was unadjusted.

Model 2 was adjusted for age, gender, education, BMI, Smoking, Drinking

Model 3 was adjusted for age, gender, education, BMI, Smoking, Drinking, hypertension, diabetes, stroke, coronary heart disease, Osteoporosis, Hyperlipidemia.

### 3.4 Non-linear relationship between WHtR and cognitive impairment

The association between WHtR and cognitive impairment was further analyzed using a restricted cubic spline model to create dose-response curves, treating WHtR as a continuous variable. Both the non-linearity test and the likelihood ratio test confirmed a significant non-linear association (both *P* < 0.001). A two-part model was employed to assess this relationship.

**Figure 2:**
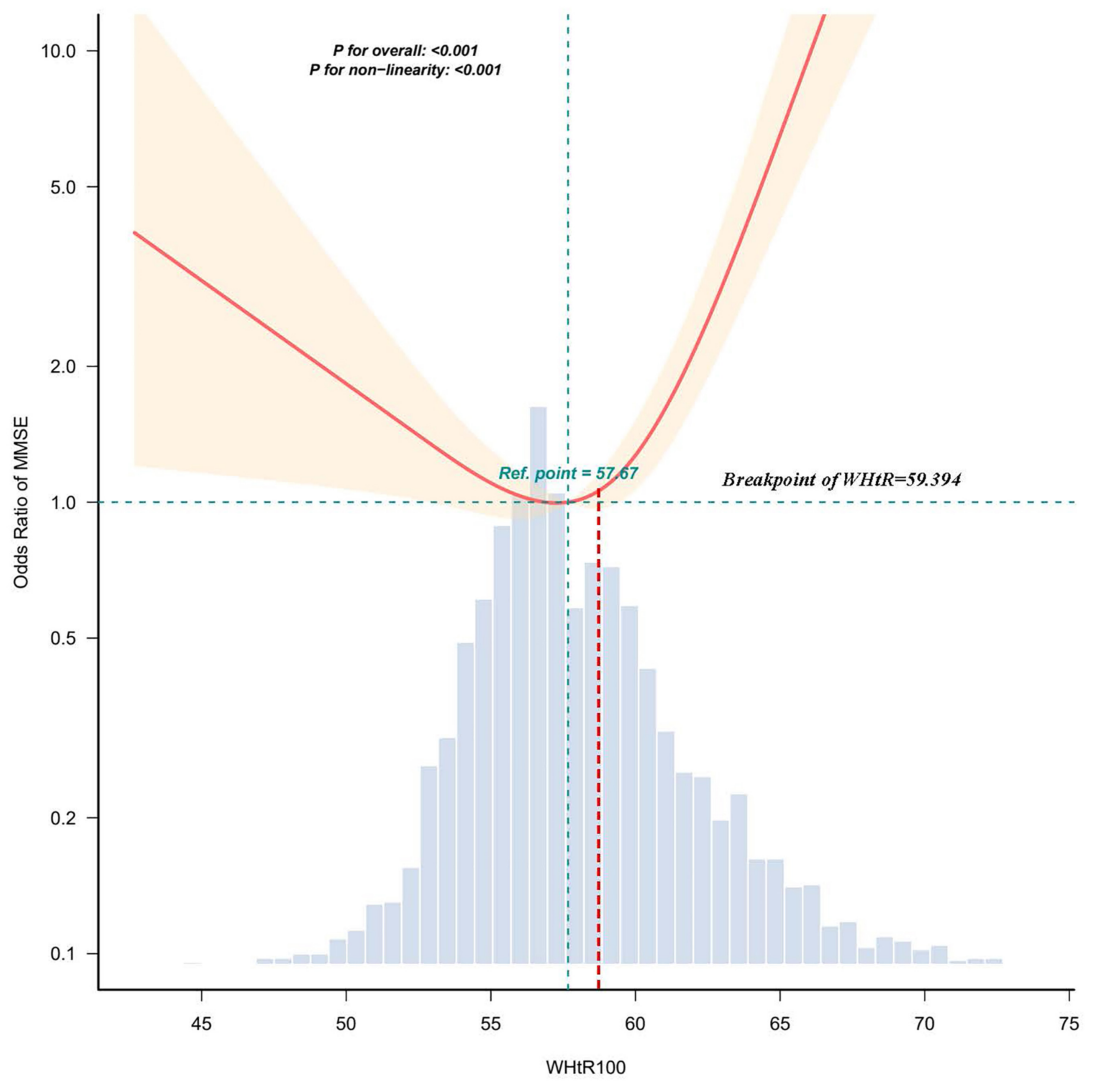
Non-linear relationship between waist-to-height ratio and cognitive impairment. The median value of 57.67 was used as the default reference point for the restricted cubic spli visualization. The clinically meaningful threshold breakpoint of 59.394 (95% CI: 59.244 to 59.544) was established through piecewise regression.

An inflection point was identified at WHtR × 100 = 59.394 (95% CI: 59.244 to 59.544). Below this threshold, no significant association was observed between WHtR and cognitive impairment (OR = 0.984, 95% CI: 0.917–1.056, *P* = 0.6513). Above this threshold, each unit increase in WHtR (multiplied by 100) was associated with a 20.8% increase in the risk of cognitive impairment (OR = 1.208, 95% CI: 1.090–1.339, *P* = 0.0003), as depicted in (Table 4, Figure 2).

**Table 4:** The non-linearity relationship between waist-to-height ratio and cognitive impairment.

Turning point of WHtR100	*N*	OR (95%CI)	*P* value
Turning point	3093	59.394 (59.244,59.544)	NA
WHtR100 < 59.394	2035	0.984 (0.917–1.056)	0.651
WHtR100 ≥ 59.394	1058	1.208 (1.090–1.339)	3e-04
Likelihood ratio test	–	–	<0.001

WHtR100, waist-to-height ratio ^*^100. Data presented are HRs and 95 CIs. Adjusted for gender, age, education level, BMI, smoking, alcohol consumption, hypertension, stroke, coronary heart disease, osteoporosis, hyperlipidemia, and diabetes.

## 4 Discussion

This community-based cross-sectional study examined the association between WHtR and cognitive impairment among elderly residents in Laoshan district, Qingdao, a representative northern coastal region of China with a mixed urban-rural population. The study revealed a non-linear association between elevated WHtR and an increased risk of cognitive impairment, with a statistically significant breakpoint identified at 59.394. Notably, the risk of cognitive impairment did not significantly increase with higher WHtR below this breakpoint; in contrast, above this threshold, each unit increase in WHtR was associated with a markedly greater risk of cognitive impairment. These findings suggest that the relationship between WHtR and cognitive impairment follows a threshold pattern rather than a simple linear trend, which aligns with recent studies reporting similar saturation or inflection point effects for central obesity indices on cognitive function.

The relationship between obesity and cognition shows inconsistency (13–15). Numerous studies have demonstrated a negative correlation between obesity and cognitive ability. An analysis of baseline data from three studies (OCTOPUS, DECS, and SuDoCo), involving a total of 1,545 participants aged 61–70 years, revealed that older adults with obesity exhibit a higher prevalence of cognitive impairment compared to their normal-weight and overweight counterparts, even after adjusting for comorbid conditions such as hypertension and diabetes (16).

Furthermore, a study utilizing panel data from the Korean Longitudinal Study of Aging (2010–2018) identified a threshold effect at a BMI of 24, indicating that increases in BMI below this threshold were associated with improved cognitive function, whereas increases above this threshold were linked to cognitive impairment among Korean middle-aged and older adults (10). Nevertheless, prior observational and longitudinal studies have shown that overweight older adults exhibit a lower risk of cognitive impairment or dementia compared to their normal-weight counterparts (15, 17, 18).

A study based on data from the 2014 baseline and 2018 follow-up of the Chinese Longitudinal Healthy Longevity Survey, which enrolled 5,156 participants aged 65 years and older, identified underweight as a significant risk factor for cognitive impairment among older Chinese adults (19). These results imply that obesity may confer a protective effect on cognitive function in older adults, often referred to as the “obesity paradox.” This paradox posits that body fat may play a protective role in specific health conditions, including cognitive function (20–22).

The reasons for these differing outcomes remain unclear. It is well recognized that BMI provides an imprecise estimate of fat mass, as it does not differentiate between types of fat or accurately reflect levels of visceral fat (23). Studies have indicated that visceral fat is more closely associated with adverse metabolic and cardiovascular outcomes than overall fat mass. Variations in body fat distribution present different patterns in relation to cognitive function (24). Visceral fat is significantly linked to insulin resistance, endothelial dysfunction, and elevated levels of pro-inflammatory cytokines, all of which are critical factors in the pathogenesis of cognitive impairment and neurodegenerative diseases (25, 26).

Therefore, it is essential to consider the impact of various anthropometric indices on cognitive impairment, particularly in contrast to previous studies that have focused on BMI as an indicator of general obesity (27). We utilized WHtR, which is emerging as a reliable and age-independent indicator of central adiposity, demonstrating greater accuracy than BMI, WC, and waist-to-hip ratio (27, 28). Our findings indicate a non-linear dose-response relationship, suggesting that the risk of cognitive impairment increases substantially with higher WHtR among older individuals. However, our study was limited by the absence of data on changes in WHtR throughout the lifespan. This limitation underscores the complexities in researching WHtR and cognitive outcomes; without long-term follow-up, prospective studies can provide only snapshots of metabolic status.

Additionally, the distribution of educational attainment varied significantly across groups, with the second quartile exhibiting the highest proportion of participants with high educational levels, whereas the fourth quartile had the highest proportion of individuals with low educational attainment. This finding indicates a higher prevalence of low educational attainment among individuals with central obesity. These observations align with studies reporting an inverse education-obesity association, demonstrating a higher prevalence of low educational status among individuals with central obesity. Education, central obesity, and cognitive function form a closely interconnected relationship (29–32). Existing evidence establishes that lower educational attainment limits health literacy and predisposes individuals to high-calorie diets and sedentary behaviors, further increasing the risk of central obesity. This is consistent with findings suggesting that poor education independently contributes to sustained BMI gain and obesity. Cross-national cohort data confirm that low education impairs cognitive reserve and is a leading modifiable risk factor for cognitive decline. When combined with the pathological effects of central obesity, individuals with lower education face a heightened vulnerability to cognitive impairment. This tripartite interaction is supported by multiple prospective studies, including those validating the inverse relationship between education and long-term BMI elevation (33).

Our cross-sectional data revealed a cognitive impairment prevalence of 54.9% among community-dwelling older adults in Laoshan, with significantly higher rates in females compared to males. This prevalence exceeds previously reported figures in regional cohorts of elderly Chinese populations (15, 34). Observed discrepancies may arise from variations in demographic composition and cognitive screening methods used across surveys. Our subgroup analyses confirmed the elevated risk of cognitive impairment in older females, consistent with multinational studies indicating poorer cognitive performance and accelerated cognitive decline in women. Participants with cognitive impairment demonstrated higher comorbidity burdens related to cardiometabolic diseases, which contribute to cerebral microvascular lesions and facilitate cognitive decline (35–38).

This study exhibits several strengths, including a large sample size, a diverse urban-rural cohort design, thorough adjustment for confounding variables, and comprehensive assessment of non-linearity through restricted cubic spline and breakpoint analysis. However, several limitations should be acknowledged. First, the cross-sectional design of the study limits the ability to draw causal inferences regarding the observed associations. Second, cognitive impairment was screened solely using the MoCA-B assessment, without formal clinical diagnostic confirmation. Third, our data were derived from a baseline survey of community-dwelling elderly adults in Laoshan, rather than hospital inpatient or outpatient clinical records. Due to the nature of community population surveys, historical imaging records of strokes were unavailable, preventing classification of stroke subtypes. Additionally, detailed information on treatment plans and control data for hypertension, diabetes, and other chronic illnesses was lacking. We aim to gradually supplement this information regarding antihypertensive therapy and blood pressure management during subsequent follow-ups of the cohort. Moreover, all cardiovascular comorbidities were identified through self-report questionnaires, which may introduce recall bias. Lastly, the sample comprised only adults aged 60–80 years, which limits the generalizability of our findings to younger age groups. Furthermore, the study did not consider other factors such as physical activity, sleep disturbances, medication use, and APOE genotype, which may result in residual confounding.

## 5 Conclusion

Among community-dwelling elderly adults in Laoshan, Qingdao, WHtR demonstrates a significant non-linear association with the risk of cognitive impairment. The risk did not significantly increase with elevated WHtR below a specific breakpoint; conversely, above this breakpoint, each unit increase in WHtR was associated with a markedly higher risk of cognitive impairment. The identified critical cutoff of WHtR = 59.394 serves as a reliable anthropometric marker for early screening and risk stratification of cognitive impairment within this regional older population. These findings suggest that maintaining WHtR within a moderate range may be beneficial for preserving cognitive health among elderly individuals in northern coastal China.

## Data Availability

The datasets presented in this study can be found in online repositories. The names of the repository/repositories and accession number(s) can be found in the article/supplementary material.
